# Vero Cytotoxin–Producing *Escherichia coli* O157 Gastroenteritis in Farm Visitors, North Wales

**DOI:** 10.3201/eid0905.020237

**Published:** 2003-05

**Authors:** Christopher J.I. Payne, Marko Petrovic, Richard J. Roberts, Ashish Paul, Eithne Linnane, Mark Walker, David Kirby, Anthony Burgess, Robert M.M. Smith, Thomas Cheasty, Geraldine Willshaw, Roland L. Salmon

**Affiliations:** * Communicable Disease Surveillance Centre (Wales), Cardiff, Wales; † Department of Public Health, North Wales Health Authority, Mold, Wales; ‡ Public Health Laboratory, Bangor, Wales; § Veterinary Laboratory Agency, Aberystwyth, Wales; ¶ Isle of Anglesey County Council, Anglesey, Wales; # Public Health Laboratory Service Central Public Health Laboratory, London, UK

**Keywords:** Case-control studies, *Escherichia coli* O157, risk factors, food handling, zoonoses, gastroenteritis, farms, research

## Abstract

An outbreak of Vero cytotoxin–producing *Escherichia coli* O157 (VTEC O157) gastroenteritis in visitors to an open farm in North Wales resulted in 17 primary and 7 secondary cases of illness. *E. coli* O157 Vero cytotoxin type 2, phage type 2 was isolated from 23 human cases and environmental animal fecal samples. A case-control study of 16 primary case-patients and 36 controls (all children) showed a significant association with attendance on the 2nd day of a festival, eating ice cream or cotton candy (candy floss), and contact with cows or goats. On multivariable analysis, only the association between illness and ice cream (odds ratio [OR]=11.99, 95% confidence interval [CI] 1.04 to 137.76) and cotton candy (OR=51.90, 95% CI 2.77 to 970.67) remained significant. In addition to supervised handwashing, we recommend that foods on open farms only be eaten in dedicated clean areas and that sticky foods be discouraged.

Human illness caused by Vero cytotoxin–producing strains of *Escherichia coli* O157 (VTEC O157) can occur after direct contact with farm animals. Although the annual rate of VTEC O157–reported illness in the general population in England and Wales is relatively low, ranging from 1.28 to 2.10 /100,000/year from 1995 to 1998 (*1*), young children who become ill are at particular risk for serious complications, such as hemolytic uremic syndrome.

An estimated half million to 10 million visits each year are made to approximately 1,000 open farms (i.e., a working farm that allows visitors, usually for an entry fee) in England and Wales (2 and Association of Farms for Schools, pers. comm.). However, only occasional outbreaks of VTEC O157 associated with such visits are reported: eight outbreaks of VTEC O157 in visitors to open farms in England and Wales were reported to the Laboratory for Enteric Pathogens at Colindale in 1992 through 2000 (*3*–*7*). The largest outbreak in the United Kingdom during this period consisted of seven cases (5). Individual cases associated with open farms are rarely reported (*8*). Of 71 reported cases of *E. coli* O157 in Wales during 1998, two primary case-patients reported visiting an open farm in the previous week (Communicable Disease Surveillance Centre, Wales, unpub. data).

Recognition of the risk of acquiring zoonotic infection, particularly gastrointestinal illness, has led to written guidelines for open farms (*9*). The guidelines concentrate on farm layout, clear routes for visitors to follow, staff training, control of animal contact, separate eating areas, adequate handwashing facilities, and adequate information in the form of notices or leaflets. These recommendations are derived from an understanding of how pathogens are likely to be spread to humans. However, the popularity of farms as a tourist attraction, when compared with the infrequency of illness, suggests that quite specific risks may occasionally occur. The opportunities for studying these risk factors more systematically are limited, as outbreaks are so infrequent. A large outbreak of gastroenteritis in North Wales, associated with VTEC O157, presented an opportunity to conduct a case-control study to investigate which factors were associated with an increased risk for illness.

## Identification, Investigation, and Control of the Outbreak

On June 9, 1999, the first report of *E. coli* in a person who had visited an open farm in North Wales was made to the communicable disease control team of North Wales Health Authority. The farm was visited and found to be operating at a generally high standard. Six days later, on June 15, two more patients with culture-positive *E. coli* O157 infection reported having visited the farm. All three case-patients had visited the farm on May 31. The farm owner immediately and voluntarily closed the farm, and all local physicians were informed of the cases by fax on June 15 and asked to report further cases.

The farm received 50,000 visitors a year and had a range of animals and several food outlets. Most contact with animals occurred in the barn, which contained a variety of farm and domestic animals. Handwashing facilities existed nearby but were not located immediately adjacent to the barn exits. Eating areas were accessible to a roaming goat. The first three case-patients had visited the farm on May 31, the 2nd day of a 2-day annual festival held on the farm. The festival had admitted 3,000 visitors each day, all of whom had access to the open farm. Attractions at the festival included food stalls and a number of visiting animals (rare and unusual farm animals brought to the farm for the festival). The working part of the farm had a sheep flock and herd of cattle.

Local case-finding efforts on June 15 did not initially disclose any further cases associated with the farm. The outbreak control team recommended washing and disinfecting all public areas and preventing contact between visitors and farm animals or animal feces. After complying with these recommendations, the farm was allowed to reopen 2 days later on June 17; however, it was to operate under a prohibition notice served under the provisions of the Health and Safety at Work Act 1974, restricting visitors from having direct contact with animals. However, later that same day a fourth case was reported in a person who had visited the farm on June 5, five days after the first three patients. The farm was formally closed to all visitors under the terms of a second prohibition notice. A national public warning was issued, all communicable disease control units were alerted, and a telephone helpline was set up and received over 150 calls. Children at three local nurseries and two primary schools, where primary cases had occurred after group visits, were screened for further cases. Health and safety arrangements were reviewed at the farm (*9*) and the recommendations of the outbreak control team were implemented; these included a one-way flow through the petting area, positioning of washing facilities immediately adjacent to the exit, exclusion of farm animals from eating areas, and reinforcement of the importance of handwashing. On June 25, the farm was allowed to reopen. Because patients reported a wide variety of activities and contact with animals, a case-control study was conducted to identify particular high-risk exposures.

## Methods

A case was defined as hemolytic uremic syndrome or *E. coli* O157 culture-positive diarrhea in any child <15 years of age who had visited the farm on or after May 31 and become ill within 10 days of the visit. Controls were selected from children <15 years old who remained well in the 2 weeks after a farm visit and whose parents had contacted the telephone helpline. Children who had visited before May 31 were excluded. Only one control child was chosen from each family or group. Where appropriate, information was obtained from an adult who had accompanied the children to the farm. Attempts to contact callers were abandoned after three separate unsuccessful day and evening phone calls.

Potential risk factors were identified from preliminary interviews, a site visit, and a risk-assessment exercise conducted by the proprietor after the outbreak was discovered. A structured questionnaire was administered by telephone to patients and controls. Questions included date of visit, contact with animals or surrounding barriers, areas of the farm visited, food consumption, personal behavior (e.g., thumb-sucking), handwashing, use of the toilet, whether soiling was visible on the child, and whether the child had fallen at the farm. In an attempt to categorize the degree of exposure to each type of animal, respondents were asked to estimate the time they spent with each. The nature of the animal contact was recorded as cuddling, kissing, feeding from hand, bottle feeding, or stroking.

The risk for illness, expressed as an odds ratio (OR), was calculated for each exposure, using Epi Info version 6 (Centers for Disease Control and Prevention, Atlanta, GA). Some exposures, such as animal contact, were analyzed both by category (“contact/no contact”) and by comparison of the risk for light contact (only feeding from the hand or bottle feeding) with more intense contact (cuddling, stroking, or kissing). To investigate confounding, logistic regression was performed, using SPSS version 7.5 for Windows (Microsoft, Redmond, WA**),** with the probability of becoming ill as the dependent variable and exposures associated with an increased risk for illness, at p<0.10, as independent variables.

Strains were confirmed biochemically and serologically as *E. coli* O157 and were phage typed and tested for resistance to antimicrobial agents by methods summarized previously (*10*). Pulsed-field gel electrophoresis (PFGE) was performed by the method of Willshaw et al. (*11*).

## Results

### Overview of Cases

A total of 17 primary cases (1 adult, 16 children) and 7 secondary cases in household contacts (2 adults, 5 children) were ascertained. Ten patients (1 adult, 9 children) required hospital admission, including 3 children with hemolytic uremic syndrome. Ten of the primary case-patients had visited the farm on May 31, Bank Holiday Monday; the remaining seven had visited during the following 15 days. No patient had visited before May 31. Isolates from all the cases except one, where the sample was not submitted for typing, were characterized as phage type (PT) 2, verocytotoxin type (VT) 2, and were resistant to streptomycin, sulphonamides, and tetracycline (SSuT). Secondary transmission was not evident in any of the schools or nurseries screened.

### Microbiology

Eleven of 46 (mainly floor) fecal samples taken by veterinarians were positive for *E. coli* O157. Ten strains were PT2, VT2, SSuT, and one strain was PT4, VT2, and sensitive to antimicrobial agents. These 11 strains were from pens or paddocks containing calves, goats, pigs, sheep, and a pony. Rabbit, fowl, and donkey samples were negative. The results of PFGE showed that the human and animal strains of *E. coli* O157 PT2 VT2 were indistinguishable.

### Case-Control Study

Sixteen children met the case definition. Questionnaires were completed for 13 of these 16 case-patients and 36 controls. Controls had a mean age of 4.5 years (SD 2.7) compared with the patients’ mean age of 5.4 years (SD 3.1); this difference was not significant (p=0.35). The proportion of girls was similar in patients (55.6%) and controls (61.5%). Table 1 shows results of univariable analysis. As all case-patients had been in contact with goats, calculating an OR for “any contact” was not possible. Goat contact was therefore stratified into high- and low-contact categories.

**Table 1:** Results of univariable analysis of risks for illness caused by *Escherichia coli* O157 in visitors to an open farm, Wales^a^

Exposure		Cases	Controls		Univariable analysis		
		Exposed/ not exposed	Exposed/ not exposed		Odds ratio	p value	95% CI
Contact with animals							
	Cows	11/2	16/20		**6.88**	**0.01**	1.15 to 52.69
	Goats (any contact)	13/0	27/9		Undef	0.09	Undef
	Goat (high contact)	10/3	17/19		**3.72**	**0.06**	0.75 to 20.70
	Rabbit	9/4	20/16		1.80	0.39	0.40 to 8.62
	Sheep	10/3	27/9		1.11	1.00	0.21 to 6.48
	Pigs	5/7	11/24		1.56	0.72	0.33 to 7.32
	Pony	4/9	20/16		0.36	0.12	0.07 to 1.61
	Shire horse	4/9	13/23		0.79	1.00	0.16 to 3.64
Areas of farm visited							
	Play area	5/7	27/9		0.24	0.07	0.05 to 1.13
	Paddock	6/7	28/8		0.24	0.08	0.05 to 1.13
	Pony ride	4/9	15/21		0.62	0.49	0.13 to 2.84
	Tractor ride	9/4	28/8		0.64	0.71	0.13 to 3.31
	Main barn	13/0	35/1		Undef	1.00	Undef
Food consumption and personal behaviors							
	Sucks thumb	1/11	6/30		0.45	0.66	0.02 to 4.74
	Bites nails	2/10	6/30		1.00	1.00	0.12to 7.08
	Ate any food while on farm	13/0	33/3		Undef	0.56	Undef
	Ate immediately after barn	6/6	11/23		2.09	0.31	0.45 to 9.81
	Ate food bought on farm	8/5	14/21		2.40	0.18	0.55 to 10.85
	Ate ice cream	9/4	14/22		**3.54**	**0.06**	0.77 to 17.19
	Ate cotton candy	7/6	4/32		**9.33**	**0.004**	1.69 to 57.10
	Bought animal feed	10/3	32/4		0.42	0.36	0.06 to 2.88
	Picked up animal feed from floor	5/8	6/30		3.13	0.13	0.61 to 16.29
	Ate animal feed	1/12	0/36		Undef	0.26	Undef
	Clung to animal barriers	6/3	25/7		0.56	0.66	0.09 to 3.76
	Fell over while on farm	1/12	7/29		0.35	0.66	0.01 to 3.43
	Washed hands at all	10/3	24/9		1.12	1.00	0.23 to 7.35
Environmental observations							
	Wet underfoot	3/10	6/30		1.50	0.68	0.24 to 8.85
	Dirty hands, shoes, or clothes	0/13	9/27		0.00	0.09	0.00 to 1.46
	Noticed queue for toilets	2/10	3/32		2.13	0.59	0.21 to 19.56
Type of visit							
	Family visit	12/1	33/3		0.92	1.00	0.08 to 30.07
	Bank Holiday Monday	11/2	13/20		**9.73**	**0.003**	1.38 to 66.32

^a^CI, confidence interval; undef, undefined. (When one of the cells contains a zero, defining a confidence interval is not possible.) Bold typeface highlights variables with increased odds ratio statistically significant at 90% level.

Attendance on Bank Holiday Monday, eating ice cream, eating cotton candy (i.e., “candy floss”), any contact with cows or goats, and high goat contact were all associated with increased risk (p<0.10). All case-patients had eaten either cotton candy or ice cream. No link between the risk for illness and duration of contact with cows or goats was found. The main barn was the only area visited by all patients.

Cotton candy was only available on Bank Holiday Monday, a special festival day on which visitors were also more likely to have contact with cows (OR 5.56, p=0.03 for those attending on May 31 compared with other days). For these reasons, Bank Holiday Monday was not an independent variable and so was excluded from the multivariable analysis. The results of multivariable analysis for the other four variables are shown in Table 2. The association between illness and eating ice cream or cotton candy increased and remained significant; the magnitude of effect for cow and goat contact was similar to univariate analysis, although neither factor was statistically significant. To check whether Bank Holiday Monday was unique, the analysis was repeated for the 24 Bank Holiday Monday attenders only; results are shown in Table 3. No OR reached statistical significance, reflecting the smaller dataset; however, the magnitude of the independent effect of these variables (as evidenced by ORs) is similar to that for the whole study population, suggesting that the risks were similar on the Bank Holiday Monday to the whole study period.

**Table 2:** Results of multivariable analysis of significant animal contact and food consumed and risk for illness (49 observations)^a^

	OR	p value	95% CI
Cows	7.19	0.07	0.86 to 59.81
Goat (high contact)	4.85	0.16	0.54 to 44.03
Ate ice cream	**11.99**	**0.046**	**1.04 to 137.76**
Ate cotton candy	**51.90**	**0.008**	**2.77 to 970.67**

^a^OR, odds ratio; CI, confidence interval. Boldface type indicates variables with increased OR statistically significant at 95% level.

**Table 3:** Results of multivariable analysis of significant animal contact and food consumed for Bank Holiday attenders only and risk for illness (24 observations)^a^

	OR	p value	95% CI
Cows	13.65	0.14	0.42 to 438.71
Goat (high contact)	3.58	0.38	0.21 to 61.98
Ate ice cream	10.35	0.10	0.63 to 169.31
Ate cotton candy	38.44	0.07	0.71 to 2,092.60

^a^OR, odds ratio; CI, confidence interval.

## Discussion

This outbreak is the largest caused by VTEC O157 in visitors to an open farm and the first case-control study of risk factors for infection on an open farm in the United Kingdom. Our study has demonstrated a strong association with eating either ice cream or cotton candy and an increase in risk associated with goat and cow contact.

A case-control study among farm visitors in the United States in 2000 showed an association between *E. coli* O157 infection and contact with cattle, nail biting, and food purchase (*12*). Handwashing was protective in that study.

Considering sources of bias, particularly in the selection of controls or in gathering information, is important. Callers to a helpline are likely to differ in some ways from the other visitors, perhaps being better informed and more anxious. However, their ice cream or cotton candy eating habits are unlikely to differ. Information on known risk factors, such as handwashing and food history, may be susceptible to “rumination bias,” that is, a tendency for those who have been ill to systematically bias the reporting of exposure. This bias would explain the apparent lack of protective effect of handwashing. Patients may have been more likely to recall eating ice cream, as this was one of the foods widely reported in the media as a possible source of infection in the early stages of the investigation. However, the association with cotton candy was unexpected, and there is no reason to think that patients were more likely to recall this than the controls.

The association between illness produced by VTEC O157 and contact with cows and goats reflects previous experience of direct transmission to humans (*4*,*5*,*7*). Cattle are regarded as the most important reservoir for VTEC O157 (*13*). However, the strong association with cotton candy and ice cream merits further discussion. Ice cream was supplied by the same local manufacturer to 65 other outlets in North Wales. Cotton candy was manufactured on site on the May bank holiday by a vendor using a process repeated at different fairs throughout North Wales. Illness associated with the ice cream or cotton candy was not reported elsewhere. However, both foods appeared to be strongly associated with the risk for illness. Both are particularly sticky, messy foods, and it is possible to envisage two mechanisms by which eating them makes the ingestion of *E. coli* O157 more likely. First, after eating one of these foods, sticky hands may be more prone to pick up contaminated organic matter from the environment or directly from animal coats by stroking. Secondly, to clean sticky hands, small children are likely to lick their fingers.

Our investigation reinforces existing advice (*9*,*14*) on handwashing, specifically that handwashing facilities should be positioned immediately adjacent to exit areas where animal contact is encouraged, and that one-way systems and adequate supervision can facilitate effective handwashing. Advice concerning the importance of supervised handwashing before and after eating should be reinforced at the point of selling food. Our findings, and those of others (*4*,*5*,*7*,*12*,*13*) also suggest that calves may not be suitable animals for petting. In addition, we recommend specifically that food, particularly sweet and sticky food, only be sold and eaten in clean areas of the farm. Ideally, such sticky foods should be discouraged altogether.
